# Temperature and cardiovascular and respiratory mortality in desert climate. A case study of Kerman, Iran

**DOI:** 10.1186/1735-2746-10-11

**Published:** 2013-01-19

**Authors:** Narges Khanjani, Abbas Bahrampour

**Affiliations:** 1 Department of Environmental Health and Department of Epidemiology and Biostatistics, School of Public Health, Kerman Medical University, Kerman, Iran; 2 Honorary Research Fellow, Monash Centre for Occupational & Environmental Health, School of Public Health and Preventive Medicine, Monash University, Melbourne, Australia; 3 Unit of Epidemiology and Biostatistics, School of Public Health, Kerman Medical University, Kerman, Iran

**Keywords:** Temperature, Mortality, Climate, Cardiovascular, Respiratory

## Abstract

Many studies have suggested that cardiovascular and respiratory disease mortality may change with fluctuations in temperature. In this study the relation between temperature and mortality has been studied in a city with desert climate. Four years data on daily temperature, cardiovascular, respiratory mortality and air pollution was acquired for Kerman, Iran. Time series, regression and correlation analyses were performed. Results showed an inverse relationship between mortality and temperature in Kerman, in which decreases in temperature were associated with increases in mortality. This pattern is similar to some foreign studies which show acclimatization of people living in southern warmer climates and less negative effects of warm temperatures. Among the pollutants only dust (p=0.003) and SO2 (p<0.001) showed a positive correlation with respiratory mortality.

## Introduction

Kerman is a city with a population of more than 677,000 (most recent census) and about 14,000 hectares surface located in the south east of Iran. The dry and desert climate (average monthly rain fall, 0.2 to 28.7 mm) is associated with a temperature difference of more than 44°C between summer (maximum 39.4°C) and winter (min −4.7°C) [1], which makes it a suitable place to study temperature related effects.

With increased greenhouse gas emissions, climate change, increased average temperatures and the world’s aging population, serious effects on human health may happen and continues to become worse in the future. Studying these changes, can help identify vulnerable populations and formulate preventive actions [2,3]. Most of the epidemiological studies about temperature and mortality come from North America and Europe. Most of these countries have had an increase in heat episodes in the past two decades, for example Europe has warmed by 0.3°C per decade since the 1970s [4]. Generally mortality is found to be lower at average temperatures and higher at low and high temperatures [5]. There is evidence of a U or V shaped relationship between mean temperature and all cause mortality in some world cities [5-7].

In the United Kingdom cold related mortality greatly increases in winter in all ages and especially among elder people [8]. A history of respiratory illness (asthma, emphysema, pneumonia and chronic cough) was a strong predictor of cold death in the elderly and was clearly associated with death from cardiovascular disease [8].

The study of Ishigami et al. in Europe and also some other studies have shown the strongest heat effects on death for respiratory and cardiovascular diseases [4]. In Ishigami’s study, data from almost all cities (except one) showed higher mortality from respiratory and cardiovascular diseases in comparison to other causes on warm days [4].

Environmental temperature not only has been studied per se but also has been reported as a major cause of fluctuations in mortality rates and may act as a confounder in other environment al and mortality studies [5]. Thus, estimating the effect of temperature can help investigators adjust for its probable effects.

Although the relationship between temperature and mortality has been examined in different cities around the world [3], and many studies of temperature and health have shown associations between temperature and cardiovascular and/or respiratory disease deaths, but a few of them were from the middle east region [9], highlighting the fact that more investigation in this area with mainly harsh desert climate is needed. This study is by our knowledge the second to study temperature and mortality in a Middle Eastern city.

## Methods

The study included four years population mortality data. Daily mortality data from 20/3/2004 to 19/3/2008 for respiratory deaths and cardiovascular deaths separately were inquired from the Kerman Province Health Authority. Also, the Weather Bureau of Kerman provided maximum and minimum daily temperature for the mentioned time frame.

Some studies have reported confounding effects for particulate matter less than 10 μm (PM10) and ozone [3]. Due to the probable confounding effect that air pollution and suspended particles may have on high or low temperature and mortality, air pollution data was obtained from the Department of Environment (DOE) of Kerman Province. Kerman has a mainly desert climate with occasional sand storms in the spring. The province’s DOE bureau routinely collects 24 hour, daily data on CO (ppm), dust (μg/m^3^), NO (ppm), NO_2_ (ppm), NOx (ppm), O_3_ (ppm) and SO_2_ (ppm). The original data was collected per hour. The mean amount of pollutants were calculated and used as 24 hour averages in our study to be able to compare with the daily mortality data. Data on humidity or influenza mortality were not accessible.

Descriptive statistics, time series analysis, regression and correlations were performed separately for cardiovascular and respiratory deaths. Analysis was performed on daily data and aggregated monthly data. The data was analyzed using MiniTab 15 and EXCEL.

Initially Box-Cox transformation was done on the data for respiratory and cardiovascular deaths. Then ACF (auto-correlation function) and PACF (partial auto-correlation function) were calculated for the time series. The ACF yields information on the correlation coefficients between lagged-series values, whereas the PACF considers the lagged correlations for the same series, controlling for the effect of the other lags [10].

## Results

Between March 20, 2004 and March 19, 2008 a total of 6456 deaths, 1619 respiratory (929 male and 690 female) and 4838 cardiovascular (2734 male, 2103 female and 1 unknown) deaths were registered in the city of Kerman; 1494 in the first year, 1590 in the second year, 1773 in the third year and 1599 in the last year . The mean number of deaths per day was 4.1, 4.4, 4.9 and 4.4 respectively from the first to the last year.

**Figure 1:**
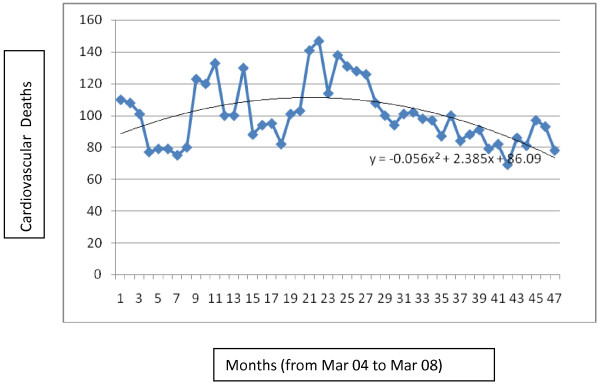
Time series of cardiovascular death in Kerman over 4 years and trend of cardiovascular death in Kerman.

**Figure 2:**
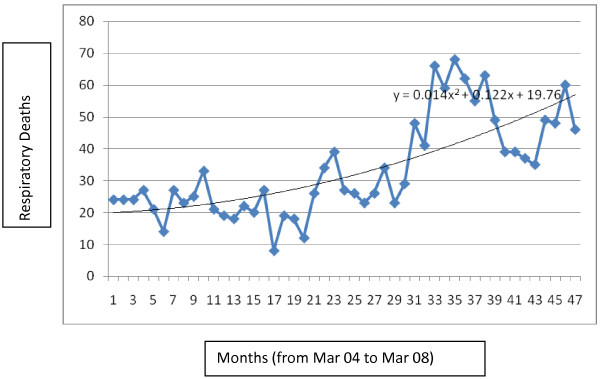
Time series of respiratory deaths in Kerman over 4 years and trend of respiratory deaths in Kerman.

The demographic description is available in Table 1. The minimum rate of mortality for both respiratory and cardiovascular deaths was during August and September, when the mean temperature were 25.1 and 21.1°C. (Figures 1, 2 and Table 1) .

**Table 1:** The mean temperature, cardiovascular and respiratory deaths in Kerman over four years

**Months**	**Mean temperature (C)**	**Minimum temperature (C)**	**Maximum temperature (C)**	**Heart disease related deaths**	**Respiratory related deaths**
January	3.8	1.9	4.9	457	186
February	7.8	6.6	10.2	412	174
March	11.2	7.0	13.4	458	150
April	17.4	17.2	17.8	425	123
May	22.0	20.2	23.7	454	132
June	26.2	24.2	27.8	406	119
July	27.6	26.6	28.0	358	127
August	25.1	24.1	26.1	* 356	* 91
September	21.1	19.5	23.1	* 324	* 99
October	16.6	14.9	18.6	363	128
November	11.2	10.1	12.0	366	125
December	5.8	3.8	7.2	459	165

* The minimum deaths in the years.

The trend of cardiovascular disease mortality is pursued in Figure 1 and the trend of respiratory disease mortality is shown in Figure 2. Different linear, quadratic and cubic models were examined for the data and eventually according to the R^2^ (crude and adjusted) second degree models were the best fit. As the graphs show crude respiratory mortality is increasing and crude cardiovascular mortality is slightly decreasing over time in Kerman.

Regression was performed for modeling mortality as a response variable to temperature, which showed slight increases in daily mortality from respiratory diseases and cardiovascular diseases as temperature decreased. Significant but weak negative correlations were observed between mortality and temperature as shown in Table 2.

**Table 2:** Correlations between mortality and temperature in Kerman

**Cause of mortality**	**Variable**	**Correlation coefficient**	**p-value**
Respiratory disease	Daily temperature	−0.157	P<0.001
	Max temperature	−0.157	P<0.001
	Min temperature	−0.146	P<0.001
Cardiovascular diseases	Daily temperature	−0.101	P<0.001
	Max temperature	−0.107	P<0.001
	Min temperature	−0.086	P=0.001

Cross-correlations (up to 40 day lags) were performed between daily cardiovascular mortality and temperature, and the maximum correlations for cardiovascular deaths were on lag 0 to lag 3 and was equal to – 0.10. The results for respiratory mortality and temperature started from – 0.15 on day 0 and reached a maximum of – 0.20 on day 26. However, the daily counts of respiratory disease in Kerman were low and on many days was zero which makes conclusions doubtful.

Correlations for 7 main pollutants (CO, dust, NO, NO2, NOx, SO2 and O3) and mortality were created. Respiratory disease mortality showed significant, but weak correlations with dust (r= 0.11, p=0.003), SO2 (r= 0.15, p<0.001) and O3 (r= − 0.13, p<0.001), but not for the other pollutants. No significant correlation was found between cardiovascular disease and the air pollutants.

Eventually based on the temperature coefficient change, the relation between respiratory mortality and temperature was adjusted for dust and SO2 as the main confounders. The temperature coefficient rose to −0.28 and was still significant (p<0.001).

**Figure 3:**
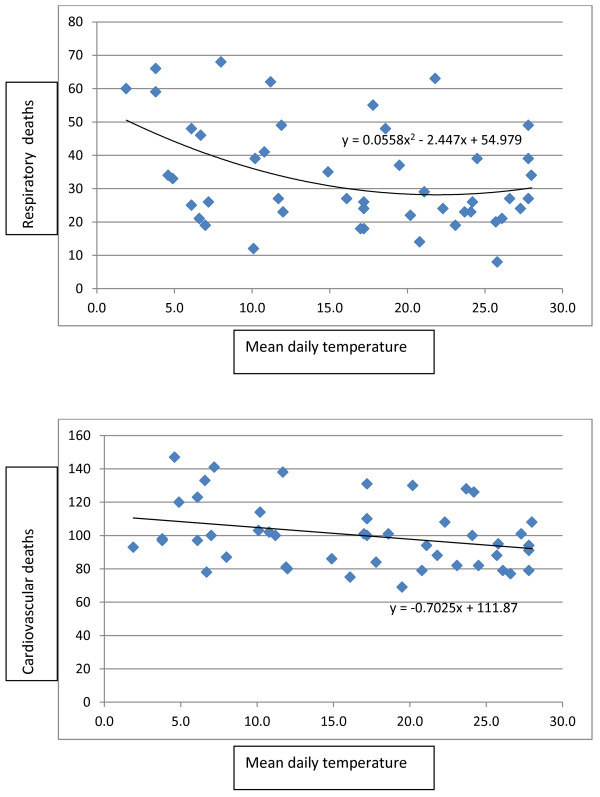
Scatterplots and fitted lines of monthly (a) respiratory disease mortality (b) and cardiovascular disease mortality and mean month temperatures.

Mortality data was aggregated for each month to avoid time points with zero mortality in daily data and scatterplots with fitted lines were created for respiratory (Figure 3-a), cardiovascular disease mortality (Figure 3-b) and mean daily temperature for the same month. The scatterplots showed an increase in mortality as temperature decreased. For each 1°C decrease in temperature, respiratory deaths showed an average of 2.5% increase in a quadratic model and cardiovascular deaths showed a 0.6% increase in a linear model. The scatterplots and fitted lines were repeated for minimum and maximum monthly temperatures, but little change was observed. Interestingly the increase in mortality associated with high and low temperatures which produced a V, U or J shaped fitted line in some cities, was not seen in Kerman which showed a more close to linear relation between mortality and temperature.

Autocorrelations in respiratory deaths were significantly correlated with lag 1, 2 and 3 and in cardiovascular deaths were correlated with lag 1 and 2 but then faded away. Partial autocorrelations were only significant for both deaths on lag 1. A 12 month cyclic pattern was the best fit for both respiratory and cardiovascular deaths.

## Discussion

Studies indicate temperature related deaths are higher for cardiovascular and respiratory deaths [11]. Our study showed an increase in cardiovascular (0.6% per°C) and respiratory (average of 2.5% per°C) mortality in Kerman with decreasing temperatures.

Many other studies have reported a delayed increase in mortality after temperature drops (cold days), lagging for a week or even more. Data from cardiovascular disease deaths from London has shown that the maximum relative risk increase of deaths for each degree C below the threshold (22.3°C) was 0.9 (95% CI 0.7 to 1.2) and occurred with a 2 to 6 day lags [5]. In Anderson et al’s study in the US heat related mortality was associated with shorter lags (same day and previous day), whereas cold related mortality was most associated with a longer lag (current and up to 25 days previous). The reason might be the fact that cold temperatures affect mortality more indirectly than heat, such as by causing infectious disease outbreaks [11]. However, Zanobetti et al. reported that lag 0 apparent temperature had the best model fit compared with the moving averages of multiple days [2].

In our study the minimum rate of mortality for both respiratory and cardiovascular deaths was during August and September. Similarly in Farajzadeh et al’s study in Tehran, the lowest number of deaths occurred in August and September. In the Tehran study the highest number of deaths had occurred in the cold months of the year (December, January, and February). Increase in cardiovascular diseases, cerebrovascular accidents, and respiratory diseases mortality all occurred during the cold months [7]. Similar to the Tehran study, in Kerman the maximum number of respiratory deaths occurred in December, January and February; for cardiovascular diseases the maximum mortality occurred during December, January and March (Table 1).

In many studies threshold effects for temperature and mortality were seen and the relation between temperature and mortality has been reported as a J or U shape graph [7,12]. The minimum mortality was observed at 16°C in the Netherlands, 27.5°C in Miami and 29°C in Taiwan. In another Iranian city, the capital Tehran with a more moderate climate, also a V-shape relationship was found between daily death numbers and daily temperature. The threshold for minimal mortality in Tehran was calculated as 28.5°C [9].

In a study in London the threshold (minimum) of total mortality and temperature was estimated to occur at about 19°C [13]. Other studies found 20°C in New Delhi, Sao Paolo, and London and 20.5°C in Christchurch [3].

Temperature threshold estimates that cold-related mortality start to increase in the range from 15.8°C to 29.8°C in different studies; and for heat-related deaths have ranged from 16.8°C to 31.8°C. Interestingly, heat thresholds were generally higher in cities with warmer climates, but cold thresholds were unrelated to climate [14].

According to the scatterplots and fitted lines in Figure 3, increase in mortality due to high temperatures was not observed in Kerman. In our study in Kerman we observed a more close to linear relationship without a threshold. Daily mortality decreased with increases in temperature and our fitted line did not have an ascending tail in warmer temperatures. The best explanation can be acclimatization of this population over the years of living in desert climate. Acclimatization can occur through physical adaptation, special housing characteristics (materials and architecture) or behavioral patterns such as staying indoors , using appropriate clothing [11] and avoiding working during the hot hours. Clothing is different from the western countries in Iran. Women wear long pants and long sleeves and cover most of their skin in public. Even for adult men short pants and sleeveless clothing is rarely used in public. Traditionally, there are still old houses made of mud and straw and wind towers in Kerman which cool the house interior.

In Anderson’s study in the United States, cold effects appeared to be larger in the south (warmer climate) than in the north (colder climate). In other words, heat effects were higher in colder communities and cold effects higher in warmer communities [11].

Similar to our results, in Anderson’s study, heat effects were generally lower in communities with higher long term temperatures. This supports the hypothesis that communities and individuals adapt to weather even during temperatures that are warm for that area. Absolute cold effects were higher in communities with higher temperature which has been also seen in previous studies [11] and our study. Anderson et al. found negligible or no effect for heat in many southern US communities [11] and similar to Kerman, some communities did not have a minimum mortality temperature [11]. Interestingly populations will undergo more adaptation to increasing temperatures, and less to decreasing temperatures [14].

Also Zanobetti et al. found a smaller risk of mortality due to high temperature in the warmer southern cities (excluding one) compared with the colder cities. Again the results were explained by the fact that persons in warmer climates tend to be more adopted to high temperature and more vulnerable to cold weather [2].

In our study, the pollutants such as dust and SO2 showed significant but weak correlations with only respiratory mortality. In Zanobetti et al’ study air pollution had no confounding effect on mortality [2]. But, in another study the authors found much higher PM10 effects on mortality during warmer days [15]. Ren et al. examined the effect of PM10 in Brisbane, Australia, and found that PM10 significantly modified the effects of temperature on respiratory and cardiovascular hospital admissions, and cardiovascular mortality at different lags [2]. Ren et al. in another study found that ozone positively modified the association between temperature and cardiovascular mortality, with stronger temperature- cardiovascular mortality associations when the ozone concentrations where higher [2].

Our study was a population based study. One limitation of our study was that we were not able to examine socioeconomic and related variables such as housing conditions and availability of air conditioning which might modify the association. Also we focused on cardiovascular and respiratory disease mortality that were the most related according to some previous literature and did not examine other specific causes of mortality that may also be related [2]. Also the effects of heat and cold on mortality may vary depending on climate factors and nonclimate factors such as diseases, sex and age [14].

Another limitation of this study is that we did not include humidity or influenza outbreaks. Although humidity and influenza have been accounted for as confounders in some studies [5], but many others have not included these variables [4,6,9].

Meanwhile, although some studies did adjust for humidity, they did not find a significant effect of humidity on mortality [3,11,12,16,17]. For example, Shumway *et al*. [18] showed that temperature, but not relative humidity, contributed significantly to mortality [9].

Also confounding by influenza epidemics, which generally occurs in the cold weather was not addressed in this study and other studies due to lack of reliable data [12,19].

Meanwhile, it has been suggested that mortality in frailer subjects is partially because of the harvesting (or mortality displacement) mechanism, in which subjects who are likely to die soon anyway, their death proceeds by only a few days or weeks by seasonal factors such as extreme winter temperatures. However few studies have addressed this issue in their analysis [20]. In this study due to the low daily mortality rate per day we aggregated mortality data to deaths per month, and were therefore not able to detect harvesting which needs a smaller time frame.

## Conclusion

The data from Kerman with a mainly desert climate showed that temperature drops in this city and possibly other cities with a similar climate are associated with an increase in respiratory and cardiovascular mortality and preventive actions should take place in cold weather. Also increases in air pollution with dust and SO2 can increase respiratory deaths and caution on polluted days is necessary.

## Competing interest

The authors declare that they have no competing interests.

## Authors’ contribution

NK: Suggested the topic, wrote the proposal, inquired the data from the holding organizations, checked the entered data, analyzed the data, and wrote the initial draft. AB: Corrected and commented on the proposal, cooperated in data entry and data analysis, corrected and commented on the final draft. All authors read and approved the final manuscript.
